# Longitudinal study of dietary patterns and hypertension in adults: China Health and Nutrition Survey 1991–2018

**DOI:** 10.1038/s41440-023-01322-x

**Published:** 2023-06-19

**Authors:** Jiguo Zhang, Wenwen Du, Feifei Huang, Li Li, Jing Bai, Yanli Wei, Zhihong Wang, Bing Zhang, Huijun Wang

**Affiliations:** Key Laboratory of Trace Element Nutrition of National Health Commission, National Institute for Nutrition and Health, Chinese Center for Disease Control and Prevention, No. 29, Nanwei Road, Xicheng District, Beijing, 100050 China

**Keywords:** Adult, Blood Pressure, Factor Analysis, Hypertension, Nutrition Surveys

## Abstract

China is undergoing the nutrition transition that may explain partly the high prevalence of hypertension. We aimed to investigate the longitudinal association between dietary patterns and hypertension in Chinese adults over 28 years of follow-up. We used data collected in the China Health and Nutrition Survey from 1991 to 2018. Adults aged 18 years and above (*n* = 15,929) were included in the analysis, for whom questionnaires and anthropometric data were collected during at least two waves. Factor analysis was conducted to derive food patterns based on 18 foods or food groups. We constructed three-level mixed-effect linear regression models to estimate systolic blood pressure (SBP) and diastolic blood pressure (DBP) in relation to quartiles of dietary pattern score and performed three-level mixed-effect logistic regression models to assess the risk of hypertension. Participants in the top quartile of the modern pattern had a decrease in SBP (β = − 0.51; 95% CI −0.86, −0.16; *P* < 0.01) when adjusted for all potential confounders, whereas participants in the top quartile of the meat pattern had an increase in DBP (β = 0.31; 95% CI 0.08, 0.53; *P* < 0.01). Participants in the highest quartile of the meat pattern were more likely to have hypertension (OR = 1.14; 95% CI 1.03, 1.24; *P* < 0.01). Adherence to the modern pattern characterized by high intake of fruits and dairy products was inversely associated with SBP, whereas the meat pattern was positively associated with DBP and the risk of hypertension. These findings may well have important public health implications.

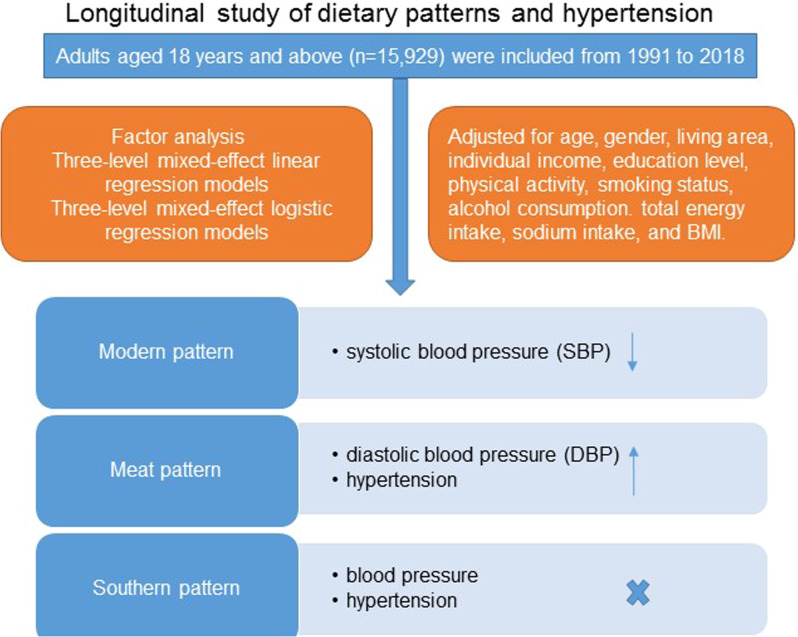

## Introduction

Cardiovascular disease (CVD) is the top leading cause of death in China, which accounted for about 44% and 47% of total deaths in urban and rural areas in 2019 respectively, according to the Annual Report on Cardiovascular Health and Diseases in China (2021) released by the National Center for Cardiovascular Diseases [1]. Among the many established risk factors for CVD, high blood pressure (BP) plays an important role and has a high prevalence of exposure [2–4]. China has experienced a growing burden of hypertension over the past decades, and the prevalence rate in adults was 27.5% in 2018 [5, 6]. The increase rate of deaths and mortality attributed to hypertension among Chinese residents were 112.72% and 77.04% respectively from 1990 to 2019 [7].

It has been recognized that unhealthy diet is an important risk factor affecting BP and dietary interventions can be effective as a strategy in the prevention and management of hypertension [8, 9]. Dietary pattern analysis has emerged as an alternative approach to provide more practical and meaningful diet information in nutritional epidemiology [10]. Several existing studies have identified various dietary patterns that are differently associated with BP and hypertension [11–13]. The Mediterranean diet (MED) and the Dietary Approaches to Stop Hypertension (DASH) diet that can have the protective effects against hypertension have been recommended and adopted as healthy dietary patterns by many countries [14–18]. However, most of the studies were performed in western communities and the results may not be capable to other populations because of the differing food culture and eating habit.

China is undergoing the nutrition transition from traditional dietary patterns to western dietary patterns, which may explain partly the high prevalence of hypertension [19, 20]. There is a need for public health professionals to provide dietary recommendation to persons who need to lower their BP or prevent development of hypertension. Previous studies have shown the association between dietary patterns and hypertension among Chinese population, which mostly were based either on cross-sectional surveys or on provincial data [21–27]. To add the substantial evidence, the current study aimed to investigate the longitudinal association between dietary patterns and hypertension in Chinese adults over 28 years of follow-up.

Point of view

- Clinical relevance
  Food-based dietary guidelines are beneficial for the prevention and treatment of hypertension.
- Future direction
  Prospective studies are needed to investigate the association of traditional dietary patterns and hypertension in Asia.
- Consideration for the Asian population
  It is necessary and important to pay attention to the influence of westernization of dietary patterns on BP among Asian populations.

## Methods

### Study population

We used data collected in the China Health and Nutrition Survey (CHNS), an ongoing large-scale, longitudinal, household-based survey initiated in 1989 and continuing in 2018. The CHNS used a multistage random-cluster process to draw the sample from the original eight provinces to final fifteen provinces: Heilongjiang, Liaoning, Beijing, Henan, Jiangsu, Zhejiang, Shanghai, Shandong, Hubei, Hunan, Shaanxi, Yunnan, Chongqing, Guizhou, and Guangxi, which varied in demography, geography, economic development, and public resources. In each province, we selected 2 cities and 4 counties by income and randomly selected 4 communities in each city or county. We randomly selected 20 households in each community. All household members were interviewed from September to November and followed in next subsequent waves. The detailed design and sampling have been reported elsewhere [28, 29]. For this study we used ten waves of the survey conducted in 1991, 1993, 1997, 2000, 2004, 2006, 2009, 2011, 2015, and 2018. Adults aged 18 years and above (*n* = 20,439) were included in the present analysis, for whom questionnaires and anthropometric data were collected during at least two waves. We excluded pregnant or lactating women (*n* = 146), those being hypertensive at baseline (*n* = 4233), and those having implausible data (*n* = 131, < 800 kilocalories [kcal] per day or > 6000 kcal for men and < 600 kcal or > 4000 kcal for women). Finally, the total number of participants and observations were 15,929 and 64,016, respectively.

The CHNS was approved by the institutional review committees of the University of North Carolina at Chapel Hill and National Institute for Nutrition and Health, Chinese Center for Disease Control and Prevention. Participants provided their written, informed consent in the survey.

### Dietary measurements

We collected diet data using weighing methods in combination with three consecutive 24 h dietary recalls (2 weekdays and 1 weekend day) in each wave of the CHNS. All condiments in the home inventory were carefully measured and recorded by trained interviewers. Standard forms was used to administer the 24 h dietary recalls in a face to face interview. The participants were asked to report the kinds and amount of the food and beverage items that they consume both at home and away from home during the previous 24 h. The average intake of the three recalls was used for each individual.

### Blood pressure measurement

BP was measured by experienced physicians using standard mercury sphygmomanometers with regular adult cuffs. Participants were told to sit in comfortable position for about 5 min before measurement. Three measurements were obtained with a 30 s interval. BP was calculated from the average of the last 2 of the 3 measurements. We defined hypertension as having a mean systolic blood pressure (SBP) ≥ 140 mm Hg or a mean diastolic blood pressure (DBP) ≥ 90 mm Hg, currently undergoing treatment with antihypertensive medication, or previously diagnosed by a physician.

### Other relevant variables

Each round of the CHNS has obtained identical questionnaires and anthropometric data with some adjustment from household members. We used a general questionnaire to collect individual and household information by face to face, including participant’s age, education, living area, smoking status, alcohol consumption, physical activity, annual household income per family member and so on. Well-trained health workers who followed a reference protocol recommended by the WHO took height, weight and other anthropometric measurements.

### Statistical analysis

We derived dietary patterns using datasets of each year combined for 18 foods or food groups (Table S1) expressed as mean intake (g/d) according to the Chinese Food Composition Table. Factors were rotated by the varimax method to achieve a more simplistic structure with greater interpretability. Which set of factors to meaningfully describe distinct food patterns was based on the eigenvalues (> 1), the scree plots, and the interpretability of the factors. Factor loadings were calculated for each food group across the factors. A factor score was calculated for each subject for each of the factors, in which intakes of 18 food groups were weighted by their factor loadings and summed.

We categorized participants into quartiles across the score of each dietary pattern. We used chi-square tests (categorical variables) and analysis of variance (continuous variables) to compare the characteristics of participants across quartiles of dietary pattern score. We constructed three-level mixed-effect linear regression models (xtmixed in STATA) to estimate SBP and DBP in relation to quartiles of dietary pattern score and performed three-level mixed-effect logistic regression models (gllamm in STATA) to assess the risk of hypertension. We calculated regression coefficients (95% CIs) and odds ratios (ORs) (95% CIs), respectively.

All statistical tests were two-tailed, and we regarded differences as significant at *P* < 0.05. For all analyses, we used SAS (Version 9.4, SAS Institute Inc., Cary, NC, United States) and Stata/SE (STATA, Version 15, StataCorp, College Station, TX, United States).

## Results

### Dietary patterns

Factor analysis revealed three dietary patterns in Table 1. Factor 1 was characterized by the food items rice, vegetables, pork, was named the southern pattern because it represents a typical traditional diet in South China. Factor 2, had high loadings for fruits, dairy products, cakes, cookies, and pastries, was called the modern pattern. Factor 3, characterized by high intakes of poultry, organ meats and other livestock meat, was thus called the meat pattern.

**Table 1:** Factor loading for dietary patterns identified by factor analysis

	Factor1	Factor2	Factor3
Rice	0.80	/	/
Vegetables	0.50	/	−0.28
Pork	0.42	/	0.31
Fish and seafood	0.30	0.25	0.25
Fast foods	−0.28	0.27	/
Other cereals	−0.48	/	/
Wheat	−0.68	/	−0.25
Fruits	/	0.62	/
Dairy products	/	0.61	/
Cakes, cookies and pastries	/	0.52	/
Eggs	/	0.44	/
Nuts and seeds	/	0.36	/
Fungi and algae	/	0.30	/
Poultry	/	/	0.42
Organ meats	/	/	0.41
Other livestock meat	/	/	0.36
Legumes	/	/	−0.27
Starchy roots and tubers	/	/	−0.57
Variance explained (%)	11.9	10.3	6.4

Absolute factor loadings ≥ 0.25 are presented for simplicity

### The characteristics of the participants

The characteristics of the participants in 1991 by quartiles of dietary pattern score are given in Table 2. We observed significant differences in age, education level, living areas, energy intake, sodium intake, physical activity and individual income among quartiles of dietary pattern score. For the southern dietary pattern, participants in the top quartile had the youngest age, the highest energy intake, the lowest body mass index (BMI), and had the lowest proportion of females, high education level, and urban residents and the highest proportion of current smokers, current alcohol drinkers, and high physical activity. For the modern dietary pattern, participants in the top quartile had the highest energy intake, highest BMI, highest SBP and DBP, and had the highest proportion of high education level, urban residents, current smokers, current alcohol drinkers and high individual income, and had the lowest proportion of high physical activity. For the meat dietary pattern, participants in the top quartile tended to be the oldest, and had the highest proportion of high education level, urban residents and high individual income, and had the lowest proportion of high physical activity.

**Table 2:** Characteristics of participants by dietary patterns in 1991

		Southern dietary pattern				Modern dietary pattern					Meat dietary pattern				
	Group 1	Group 2	Group 3	Group4	*p*	Group 1	Group 2	Group 3	Group4	*p*	Group 1	Group 2	Group 3	Group 4	*p*
*N*	1282	1282	1283	1282		1282	1282	1283	1282		1282	1282	1283	1282	
Age (years), mean ± SD	39.8 ± 14.1	40.8 ± 14.6	40.1 ± 13.5	38.2 ± 12.1	< 0.0001	40.5 ± 14.0	40.1 ± 13.8	39.0 ± 13.4	39.2 ± 13.3	0.0206	38.9 ± 12.6	39.1 ± 13.3	40.4 ± 14.4	40.5 ± 14.2	0.0034
Female, %	55.3	60.9	53.3	43.8	< 0.0001	53.7	54.4	54.6	50.6	0.1379	51.2	53.5	56.6	52.0	0.3846
Senior high school and above, %	14.1	21.8	16.1	12.1	< 0.0001	9.4	11.1	17.2	26.4	< 0.0001	10.9	12.4	16.9	23.8	< 0.0001
Urban, %	28.9	45.9	33.4	17.2	< 0.0001	17.9	28.2	35.2	44.2	< 0.0001	17.9	21.4	35.4	50.9	< 0.0001
Current smoker, %	33.8	29.1	32.9	40.0	0.0002	33.0	33.5	32.0	37.3	0.0443	36.2	34.9	30.9	33.8	0.0581
Current alcohol drinker, %	36.8	34.6	38.9	42.3	0.0006	35.9	35.8	37.2	43.7	< 0.0001	40.4	38.5	36.1	37.5	0.0673
High individual income, %	27.3	42.0	35.4	28.4	< 0.0001	16.0	28.8	38.6	49.7	< 0.0001	25.3	26.6	33.2	48.0	< 0.0001
High physical activity, %	34.7	21.6	32.4	45.4	< 0.0001	48.4	37.3	27.9	20.4	< 0.0001	39.9	41.9	32.2	20.0	< 0.0001
Energy intake (kcal/day), mean ± SD	2437.8 ± 706.7	2156.1 ± 691.3	2348.2 ± 613.3	2705.4 ± 635.9	< 0.0001	2393.0 ± 675.0	2271.6 ± 699.5	2387.7 ± 630.5	2595.3 ± 719.4	< 0.0001	2670.4 ± 664.1	2463.6 ± 648.4	2228.3 ± 661.1	2285.4 ± 704.7	< 0.0001
Sodium intake (mg/day), mean ± SD	7356.0 ± 4209.7	6945.5 ± 3973.8	7098.5 ± 3762.0	7303.1 ± 3812.9	0.0310	6855.2 ± 3927.7	7193.5 ± 3843.0	7328.6 ± 3875.6	7325.7 ± 4116.6	0.0065	7709.2 ± 3937.1	7174.6 ± 3903.9	6817.7 ± 3888.0	7001.8 ± 4001.2	< 0.0001
BMI (kg/m^2^), mean ± SD	22.2 ± 2.8	21.5 ± 2.6	21.2 ± 2.5	20.9 ± 2.2	< 0.0001	21.2 ± 2.5	21.3 ± 2.6	21.5 ± 2.6	21.8 ± 2.7	< 0.0001	21.4 ± 2.4	21.4 ± 2.6	21.5 ± 2.7	21.5 ± 2.7	0.4075
SBP (mm Hg)	111.9 ± 11.7	110.0 ± 11.9	108.8 ± 11.7	109.0 ± 11.5	< 0.0001	109.5 ± 11.8	109.3 ± 11.6	109.7 ± 11.5	111.1 ± 12.1	0.0006	110.2 ± 12.1	109.3 ± 11.5	109.9 ± 11.6	110.2 ± 11.9	0.1520
DBP (mm Hg)	72.8 ± 8.1	71.5 ± 8.2	70.9 ± 8.5	70.9 ± 8.2	< 0.0001	71.2 ± 8.3	71.0 ± 8.5	71.6 ± 8.1	72.4 ± 8.1	0.0003	71.6 ± 8.3	71.5 ± 8.3	71.5 ± 8.2	71.6 ± 8.4	0.9876

ANOVA for continuous variables and χ^2^ for categorical variables

### Dietary Patterns and BP/ Hypertension

Table 3 shows the longitudinal association of dietary patterns with SBP and DBP. Participants in the top quartile of the modern pattern had a decrease in SBP (β = −0.51; 95% CI −0.86, −0.16; *P* < 0.01) when adjusted for all potential confounders. The meat pattern was significantly associated with higher DBP (β = 0.31; 95% CI 0.08, 0.53; *P* < 0.01) when adjusted for all potential confounders.

**Table 3:** Regression coefficients (95% CI) of SBP and DBP according to dietary patterns^*†^

	Q1	Q2	Q3	Q4
SBP				
Southern pattern				
Model 1	0.00 (ref)	−0.30 (−0.67, 0.08)	−0.18 (−0.59, 0.24)	−0.11 (−0.54, 0.32)
Model 2	0.00 (ref)	−0.30 (−0.67, 0.07)	−0.17 (−0.59, 0.24)	−0.14 (−0.58, 0.30)
Modern pattern				
Model 1	0.00 (ref)	−0.07 (−0.37, 0.23)	−0.07 (−0.38, 0.24)	−0.50 (−0.84, −0.14)**
Model 2	0.00 (ref)	−0.09 (−0.38, 0.21)	−0.11 (−0.42, 0.20)	−0.51 (−0.86, −0.16)**
Meat pattern				
Model 1	0.00 (ref)	0.05 (−0.25, 0.35)	0.25 (−0.06, 0.57)	0.12 (−0.22, 0.46)
Model 2	0.00 (ref)	0.08 (−0.22, 0.38)	0.27 (−0.05, 0.58)	0.08 (−0.25, 0.42)
DBP				
Southern pattern				
Model 1	0.00 (ref)	−0.13 (−0.38, 0.11)	−0.10 (−0.38, 0.17)	0.08 (−0.21, 0.37)
Model 2	0.00 (ref)	−0.13 (−0.38, 0.12)	−0.13 (−0.40, 0.15)	−0.00 (−0.30, 0.29)
Modern pattern				
Model 1	0.00 (ref)	0.29 (0.09, 0.49)**	0.23 (0.01, 0.44)*	−0.08 (−0.30, 0.15)
Model 2	0.00 (ref)	0.27 (0.07, 0.47)**	0.17 (−0.03, 0.38)	−0.13 (−0.36, 0.09)
Meat pattern				
Model 1	0.00 (ref)	−0.02 (−0.21, 0.18)	0.04 (−0.17, 0.25)	0.33 (0.09, 0.55)**
Model 2	0.00 (ref)	0.02 (−0.17, 0.22)	0.08 (−0.13, 0.29)	0.31 (0.08, 0.53)**

^*^P < 0.05, ^**^P < 0.01

^*^All of the models were constructed using three-level mixed-effects linear regression

^†^Model 1 adjusted for age, gender, living area, individual income, education level, physical activity, smoking status, alcohol consumption, antihypertensive medication usage. Model 2 additionally adjusted for total energy intake, sodium intake and BMI

Table 4 shows overall association between the dietary patterns and the risk of hypertension. After adjusting for all potential confounders, participants in the highest quartile of the meat pattern were more likely to have hypertension (OR = 1.14; 95% CI 1.03–1.24; *P* < 0.01). We observed no significant association between the traditional southern pattern and modern pattern and risk of hypertension.

**Table 4:** ORs (95% CI) of hypertension across dietary patterns^*†^

	Q1	Q2	Q3	Q4
Southern pattern				
Model 1	1.00 (ref)	0.98 (0.88,1.07)	0.95 (0.85,1.06)	0.93 (0.83,1.04)
Model 2	1.00 (ref)	0.99 (0.90,1.09)	0.98 (0.87,1.09)	0.95 (0.84,1.06)
Modern pattern				
Model 1	1.00 (ref)	1.01 (0.93,1.10)	1.01 (0.92,1.09)	0.95 (0.86,1.04)
Model 2	1.00 (ref)	1.00 (0.92,1.08)	0.99 (0.91,1.07)	0.94 (0.85,1.03)
Meat pattern				
Model 1	1.00 (ref)	1.09 (1.01,1.18)*	1.07 (0.98,1.17)	1.13 (1.03,1.23)**
Model 2	1.00 (ref)	1.11 (1.02,1.19)*	1.09 (1.00,1.19)*	1.14 (1.03,1.24)**

^*^P < 0.05, ^**^P < 0.01

^*^All of the models were constructed using three-level mixed-effects logistic regression

^†^Model 1 adjusted for age, gender, living area, individual income, education level, physical activity, smoking status, alcohol consumption. Model 2 additionally adjusted for baseline SBP, baseline DBP, total energy intake, sodium intake and BMI

## Discussion

In this large-scale, 28-year follow-up study, we found that meat pattern was positively associated with DBP and the risk of hypertension among Chinese adults independent of potential confounding factors. Furthermore, participants with higher adherence to the modern pattern had significantly lower SBP. There was no significant association between the southern pattern and BP and hypertension. To the best of our knowledge, this is the longest cohort study to examine the longitudinal association between dietary patterns and BP as well as the risk of hypertension.

Over the last two decades, the meat pattern has become more prevalent in China [19]. The meat pattern also called animal pattern has been considered to be an unhealthy dietary pattern, which is most common and characterized by a high intake of red and processed meat in western countries [30]. The meat pattern in our study was a blend of healthy (poultry, fish and seafood) and unhealthy (red meat) constituents of dietary patterns. However, our findings were consistent with previous studies, which reported a significantly positive association between meat consumption and the risk of hypertension [31–33]. Moreover, we found meat pattern was positively associated with DBP, which was in agreement with previously reported findings of similar dietary patterns [34, 35]. This relationship was perhaps due to the fact that the fatty pork was the main component of meat intake among Chinese population [36]. There are several potential mechanisms through which the intake of meat could had adverse impact on blood pressure: the joint effect of multiple dietary components, such as saturated fat and cholesterol [32]; Millard reaction products in cooked meat through its inflammatory and oxidation pathways [31]; high concentrations of nitrite additives for food preservation in processed meat [33].

Our findings showed a negative association between the modern dietary pattern and SBP. Similar to our results, a cohort study of middle-aged and elderly men in Shanghai suggested that the ‘fruit and milk’ pattern was significantly associated with both lower SBP and DBP [34]. Besides, a community–based survey in Eastern China found that the fruit-dairy pattern was inversely associated with the risk of hypertension among the study participants [27]. The modern pattern in our study had high loadings for fruits, dairy products, eggs and nuts, all of which have been reported to have beneficial effects on BP [31]. This pattern also had high loadings for cakes, cookies and pastries, the deleterious effect of which may be counteracted by the protective effect of other food groups. However, synergistic effects of dietary patterns on BP are still unclear exactly.

The southern pattern that was the predominant dietary pattern in China, did not significantly affect BP and the risk of hypertension. This null association observed in our study was consistent with a previous report in a city of Eastern China [27]. Similarly, a traditional pattern with high loading of cereals and vegetables was not related to the risk of hypertension among Korean women aged 30–79 years [37]. Lack of association may be due to the interactions among different food groups. High intake of refined cereal and pork may exert opposite unfavorable effects on BP in spite of the high level of vegetable consumption. Our previous study found that the southern pattern was inversely associated with risk of overweight/obesity [38], which was considered as important risk for hypertension [20]. Thus, the southern pattern may have an indirectly beneficial role in the prevention of hypertension.

### Asian perspectives

Some studies in Asia indicated that the healthy dietary patterns are associated with a decreased odds of hypertension [13]. However, further prospective studies are needed to better understand the influence of westernization of dietary patterns on BP among Asian populations.

There were some limitations in our study. First, the study subjects may not be representative of the whole Chinese population. Second, factor analysis involves a series of subjective decisions which may affect interpretation and it is difficult to compare results across studies because of differences in the dietary assessment methods, the number and type of food groupings. Third, the 24 h dietary recall method cannot reliably reflect usual dietary intake. However, the current study includes a relatively large sample size across a wide age range during a long time, and provides useful information to substantially support previous studies by examining the longitudinal relationship of dietary patterns with BP and the risk of hypertension.

In conclusion, adherence to the modern pattern characterized by high intake of fruits and dairy products was inversely associated with DBP, whereas the meat pattern was positively associated with SBP and the risk of hypertension. These findings may well have important implications for public health practice, not only for demonstrating the important roles of some specific food groups on BP and hypertension, but also for adding the evidence for the development of practical food-based dietary guidelines for different populations.

### Supplementary Information

Table S1
