# Supplementary material for: Longitudinal study of dietary patterns and hypertension in adults: China Health and Nutrition Survey 1991–2018
Source: Hypertens Res. 2023 Jun 19;46(10):2264–71. doi: 10.1038/s41440-023-01322-x (PMC10550817; doi:10.1038/s41440-023-01322-x)
Supplement: Supplementary file 1 — Table S1 [file 41440_2023_1322_MOESM1_ESM.docx]

Table S1 Food groups in the factor analysis

| Food or food groups | Foods included in the group |
| --- | --- |
| Rice | Round grained rice, long grained rice, and products |
| Wheat | Wheat flour and products |
| Other cereals | Corn, barley, millet, and products |
| Starchy roots and tubers | Potato, sweet potato, starch, and products |
| Legumes | Soybean, and products |
| Fungi and algae | Mushroom, kelp, laver |
| Vegetables | Cabbage, eggplant, carrot, pepper, lettuce, other vegetables |
| Fruits | Apple, pear, peach, date, grape, watermelon, orange, other fruit |
| Pork | Pork and pork products |
| Other livestock meat | Beef, game, lamb, and meat products |
| Poultry | Chicken, duck, goose |
| Organ meats | Organ meats |
| Fish and seafood | Fish, shrimp, crab, shellfish |
| Dairy products | Milk, yogurt and products |
| Eggs | Eggs and products |
| Nuts and seeds | Walnut, almond kernel, peanut, and others |
| Cakes, cookies and pastries | Cakes, cookies and pastries, bread, biscuit |
| Fast foods | Convenience food, hamburger, pizza, sandwich, french fries |
